# Supplementary material for: Evaluation of spatial Bayesian Empirical Likelihood models in analysis of small area data
Source: PLoS One. 2022 May 27;17(5):e0268130. doi: 10.1371/journal.pone.0268130 (PMC9140259; doi:10.1371/journal.pone.0268130)
Supplement: S1 File — (PDF) [file pone.0268130.s001.pdf]

Appendices

Appendix 1

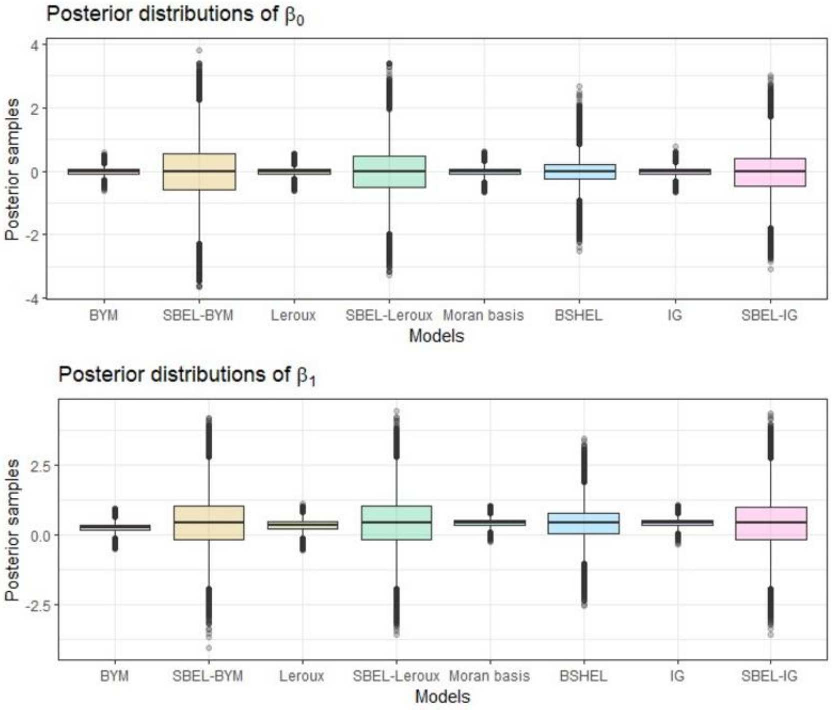

Fig.A1: Boxplots of Posterior distributions of regression coefficients (parametric vs semi-parametric) for Scottish lip cancer data

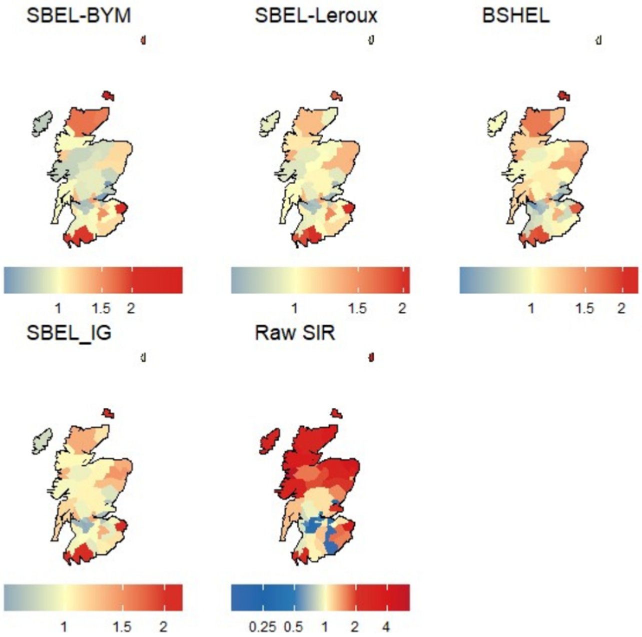

Fig.A2: Raw and Smoothed SIRs of lip cancer in males for each county in Scotland applying spatial BEL models

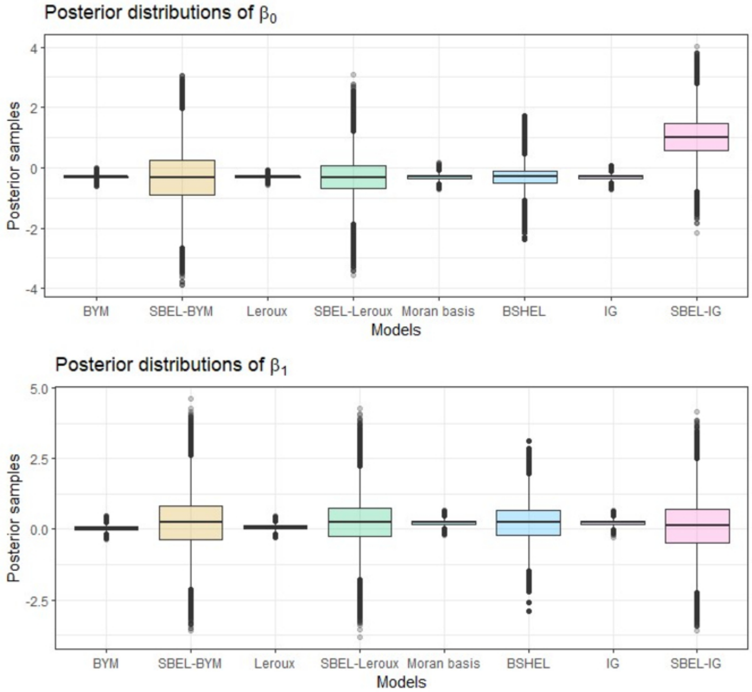

Fig.A3: Boxplots of Posterior distributions of regression coefficients (parametric vs semi-parametric) for North Carolina SIDS data

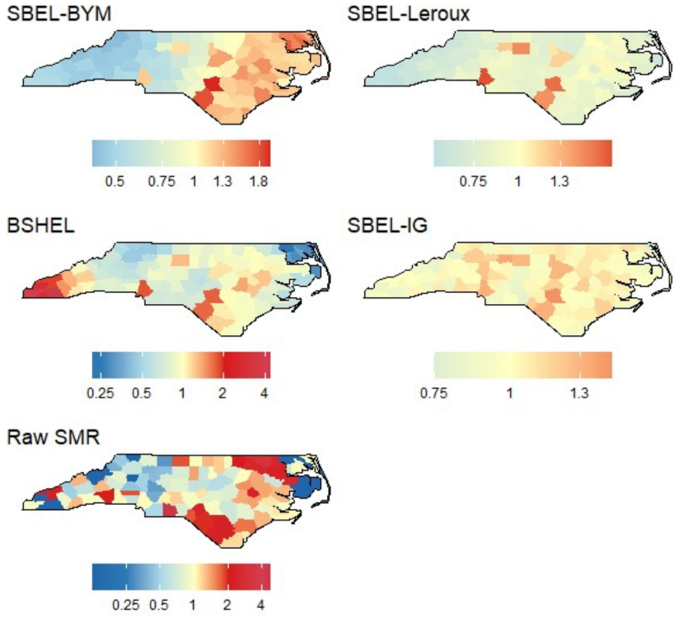

Fig. A4: Raw and Smoothed SMRs from SIDS for each county of North Carolina applying spatial BEL models

Table A1: Posterior Summaries of regression coefficients ($\boldsymbol{\beta}$) and precision parameter (τ) for COVID 19 Data using SBEL and Bayesian parametric Models

	Models	Parameters	Mean (95% CI) Jan-Apr	May-Aug	Sep-Dec
SBEL Models	SBEL-BYM	β_0	-8.99 (-9.59,-8.42)	-10.22 (-10.72 ,-9.66)	-7.50 (-8.42, -6.37)
		β_1	0.64 (0.13,1.15)	0.65 (0.20,1.08)	0.68 (0.19 , 1.14)
		τ	0.04 (0.03,0.06)	0.038 (0.026, 0.056)	0.037 (0.026, 0.057)
	SBEL-Leroux	β_0	-8.73 (-10.13,-6.31)	-10.12 (-10.92,-8.81)	-7.33 (-9.27, -4.24)
		β_1	0.58(0.29,0.83)	0.64 (0.43, 0.84)	0.64 (0.31,0.93)
		τ	0.037 (0.024,0.052)	0.04 (0.025,0.054)	0.035 (0.024,0.054)
	BSHEL	β_0	-8.91 (-10.47,-5.69)	-10.90 (-10.9031,-10.9029)	-7.32 (-9.76,-3.57)
		β_1	0.56 (0.05,0.94)	0.39 (0.393847, 0.393859)	0.64 (0.14, 1.10)
		τ	0.028 (0.006,0.168)	0.087 (0.01,0.47)	0.021 (0.004,0.122)
	SBEL-IG	β_0	-8.92 (-10.07, -6.72)	-10.14 (-10.88, -9.04)	-7.36 (-9.09, -4.68)
		β_1	0.59 (0.35,0.81)	0.65 (0.45, 0.84)	0.64 (0.35, 0.90)
		τ	0.04 (0.03,0.05)	0.037 (0.025,0.054)	0.03 (0.0254,0.055)
Parametric	BYM	β_0	-8.38 (-9.84, -6.91)	1.23 (-6.23,8.73)	-6.75 (-8.18,-5.35)
		β_1	0.56 (0.45,0.67)	0.90 (0.42,1.39)	0.61 (0.50,0.73)
		τ	0.041 (0.027,0.059)	0.015 (.003,0.374)	0.039 (0.025, 0.059)
	Leroux	β_0	-8.93 (-10.83,-6.41)	2.81 (-4.30, 9.92)	-7.09 (-8.45,-5.67)
		β_1	0.60 (0.48, 0.73)	0.64 (0.53, 0.76)	0.62 (0.50, 0.74)
		τ	0.049 (0.032,0.072)	0.01 (0.002, 0.092)	0.039 (0.025,0.059)
	Moran basis	β_0	-8.93 (-10.84,-7.02)	-10.20 (-11.93,-8.48)	-7.38 (-9.38 -5.39)
		β_1	0.61 (0.48,0.73)	0.65 (0.53,0.77)	0.65 (0.51, 0.78)
		τ	0.064 (0.04,0.092)	0.07 (0.05, 0.102)	0.053 (0.035, 0.077)
	IG	β_0	-8.94 (-10.11,-6.67)	2.82 (-4.35, 9.96)	-7.39 (-9.34 -5.44)
		β_1	0.61 (0.48,0.73)	0.84 (0.36, 1.33)	0.65 (0.52, 0.78)
		τ	0.067 (0.044, 0.946)	0.009 (0.002, 0.091)	0.056 (0.037 , 0.085)

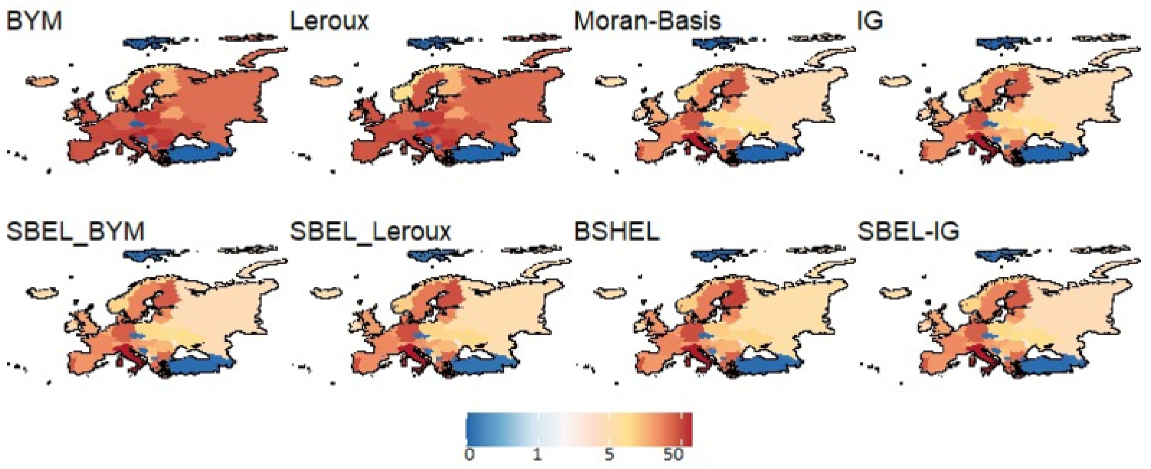

Fig.A5: Smoothed new deaths per million for Europe in Sep-Dec, 2020.

Appendix 2: Choice of spatial dependence parameter for SBEL-Leroux Model

The proposed SBEL-Leroux model involves choice of the spatial dependence parameter ρ. The spatial dependence parameter ρ can take any values between 0 (denotes no spatial autocorrelation among the small areas) and 1 (intrinsic autoregression among the small areas) [1]. In the proposed spatial BEL models using Leroux CAR prior for spatial random effects, the choice of ρ is made by comparison of model performance using WAIC [2].

In the present study, ρ is chosen to be 0.5 for the Scottish lip cancer data and 0.9 for the North Carolina SIDS data on the basis of model performance (Figure A5). It can be observed that model performance of the SBEL-Leroux model varies with the choice of ρ. So appropriate choice of the spatial dependence parameter for each data set before fitting the spatial BEL model with Leroux CAR structure prior will result in better model performance.

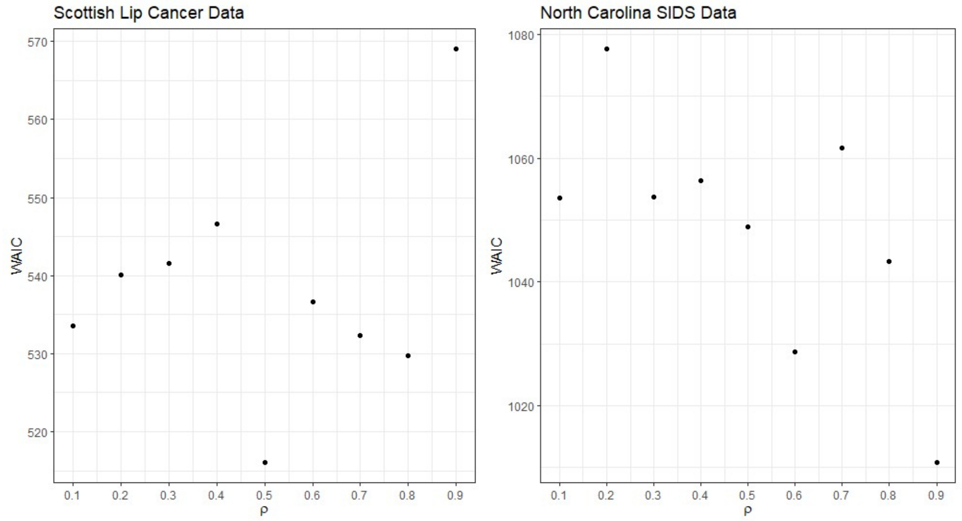

Fig. A6: WAIC of SBEL-Leroux model fitted to Scottish lip cancer and North Carolina SIDS dataset (iterations= 10000)

Appendix 3: Data Sources

All the datasets used in this study are secondary datasets. The links to access the datasets are given in this section. These data sets are publicly available and no specific rights are required to access these datasets.

1. **Scottish Lip Cancer Data:** `https://geodacenter.github.io/data-and-lab/scotlip/`
2. **North Carolina SIDS data:** `https://r-spatial.github.io/spdep/articles/sids.html`
3. **COVID19 data:** `https://github.com/owid/covid-19-data/tree/master/public`

References

1. Leroux BG. Modelling spatial disease rates using maximum likelihood. Statistics in Medicine. 2000;19(17-18):2321–2332.

2. Watanabe S. Asymptotic equivalence of Bayes cross validation and widely applicable information criterion in singular learning theory. Journal of Machine Learning Research. 2010;11(12).
