# Supplementary material for: Evaluation of spatial Bayesian Empirical Likelihood models in analysis of small area data
Source: PLoS One. 2022 May 27;17(5):e0268130. doi: 10.1371/journal.pone.0268130 (PMC9140259; doi:10.1371/journal.pone.0268130)
Supplement: Attachment — Submitted filename: Referee_Report_PONE-D-21-18812.pdf [file pone.0268130.s002.pdf]

Referee report for "Evaluation of spatial Bayesian Empirical Likelihood models in analysis of small area data"

Summary

This article applies Bayesian empirical likelihood (BEL) models to spatial data analysis for small area estimation. Simulation study and analysis of real data have been considered.

Comments

This article is poorly written. It is unable to deliver the main idea of the proposed model that employs the empirical likelihood (Section 2.1), so it seems that the proposed model is a regular Bayesian model with implementation with Gibbs sampler (Section 3.2).

1. The recaps (Section 2) are poorly written, this section is not able to point out the main idea of the empirical likelihood. I think the authors might want to skip the whole section. Here for example in page 3 line 69, it mentions that $w_i = \frac{1}{n}$ for all i, not sure whether it is the same as in [24], but in line 83, it suggests that this w_i could be found in [24] and not sure how we could derive from the equations in line 80. Furthermore, in equation 1, w_i turns out to be $w_i(\theta)$, and not sure how this connects to the previous equations in line 80. And in equation 2, the w_is now are not related to θ. So, I am not sure how the readers could go further in next section.
2. This article focus on the spatial model and here I could only see the priors available in section 3. Where I could see the spatial effect contributed by the empirical or it is just a simple extension?
3. Here I could see the likelihood in equations 23, 24 I guess, that is $p(Y|\psi, \beta)$ and is it a component in the empirical likelihood?
4. In the simulation study, I am not sure why the posterior median are given, not posterior mean (commonly used) in Tables 1, 2.
5. I think the contribution of this article is quite limited since it is not able to deliver the main scope of the empirical likelihood.
