# Supplementary material for: Evaluation of spatial Bayesian Empirical Likelihood models in analysis of small area data
Source: PLoS One. 2022 May 27;17(5):e0268130. doi: 10.1371/journal.pone.0268130 (PMC9140259; doi:10.1371/journal.pone.0268130)
Supplement: Attachment — Submitted filename: Response to Reviewers_FJ.pdf [file pone.0268130.s003.pdf]

Editor's comments:

Is there any biological insight resulted from your COVID-19 data analysis?

Response:

The analysis performed with COVID-19 data was not directly related to any biological insights, but it provided methods that can enable us to validate few assumptions using the data and model outcomes. For example, from the very beginning of pandemic we are listening to the facts that COVID 19 is more severe in elderly people and immunocompromised people. In the analysis conducted in this article, we modelled COVID-19 deaths using the covariate proportion of population aged 65 and over in each of the 54 countries in Europe during 2020 (over three periods). The model revealed significant positive coefficient of the regression which in a way validated the assertion of more COVID-19 deaths on the average to the populations with higher proportion of elderly people. The proposed model was able to model the data irrespective of the irregularities and non-normality of the response variable and enabled to validate assertion with support of data. This analysis conducted is just an indication of how spatial BEL models applied to COVID 19 or similar data with irregularities can validate multiple research questions.

The above is also added to the discussion section of the paper:

> This is to note that, the analysis performed with COVID-19 deaths data was not directly related to any biological insights, but it provided methods that can enable us to validate few assumptions using the data and model outcomes. For example, from the very beginning of pandemic we are listening to the facts that COVID-19 is more severe in elderly people and immunocompromised people. In the analysis conducted in this article modelled COVID-19 deaths using the covariate proportion of population aged 65 and over in each of the 54 countries in Europe during 2020. The model revealed significant positive coefficient of the regression which in a way validated the assertion of more COVID 19 deaths on the average to the populations with higher proportion of elderly people. The proposed model was able to model the data irrespective of the irregularities and non-normality of the response variable and enabled to validate assertion with support of data. This analysis conducted is an indication of how spatial BEL models applied to COVID 19 deaths or similar data with irregularities can validate multiple research questions.

Response to Reviewer 1:

Reviewer #1:

Evaluation of spatial Bayesian Empirical Likelihood model in analysis of small area data

The authors proposed Bayesian empirical likelihood models with considerations of different spatial priors to analyze the spatial datasets at small area level and provided the comparison with (semi-)parametric spatial models in this study.

Their simulation study or real data analysis demonstrated the BEL models can outperform the parametric spatial models once the assumption of distribution of data is validated. But when analyzing the data, you might consider a reasonable distribution for the data not just only assuming the normality. So I'm wondering if the performance of

parametric models will be reversed (better than BEL) when you consider a reasonable model (distribution, that is Poisson, negative binomial, etc.). I admit that it makes computational complex but it could be worth some discussion.

Response:

The simulated datasets were generated from normal distribution under different scenarios (small number of areas, larger number of areas, including mixture of two normal distribution and outliers). There was deviation from normality in the resulting data was due to the reasons of small number of areas, presence of outliers and mixture. But the data values were continuous, hence we could not use Poisson or Negative Binomial distribution to model the simulated outcome data.

It is mentioned in the discussion that if the response data follows any parametric distribution reasonably well (for count type data it can be Poisson or Negative Binomial, for continuous, any continuous distribution, such as Normal distribution), parametric models will be better suited. This is also shown in the analysis of Scottish Lip cancer data and North Carolina SIDS data:

> The comparison of the model performances shows that parametric spatial models are a better choice for situations in which the underlying parametric assumptions are satisfied as reflected by the model performance in the case studies using Scottish Lip cancer data and North Carolina SIDS data. However, the results of the case studies suggested that the estimated regression coefficients of spatial BEL models were similar to those estimated by parametric spatial models with wider credible intervals, which is expected as the BEL models were using the empirical likelihood of the data to obtain the posterior without imposing any distributional assumptions on the data.

Regarding to model selection, could they consider some other approaches for selection such as the K-fold cross-validation utility (Piironen and Vehtari, 2017) to evaluate the quality of the predictive distribution of the candidate model with the marginal predictive likelihood (MPL).
Piironen, J. and Vehtari, A. (2017). Comparison of bayesian predictive methods for model selection. Statistics and Computing, 27(3):711–735.

Response:

Thanks for the reference that discusses comparison of Bayesian predictive methods for model selection. The reference paper discusses some criteria for model selection using K fold cross validation, information criteria such as DIC, WAIC, leave one out cross validation among others. All the model selection criteria described in the paper are described for Bayesian model but without any spatial component, that is for non-spatial models. The reason of not considering any of these cross-validation techniques for model selection is that the spatial component of the model adds an additional objective that often competes with goodness-of-fit. That is, goodness-of-smoothing and goodness-of-fit criteria will often lead to different "best" models. Additionally, in a spatial setting for small area level data, leaving out one or more small areas from the model as in cross validation would completely change the neighbourhood patterns, and underlying

spatial dependence. Thus the goodness of fit and model selection for spatial modelling needs different considerations as mentioned in Duncan and Mengersen (2020). So we followed the recommendations of Duncan and Mengersen (2020) and chose WAIC as the model selection criteria, as it is also showed valid for spatial models. While this criteria is not perfect for spatial models, more research needs to be conducted to find out ways to apply cross validation or other selection criteria for Bayesian spatial models in small area level, which is beyond the scope of this current paper.

This has now been added to the section 3.4 of the paper:

> It is to be acknowledged that there are other available model selection criteria such as K-fold cross validation, information criteria such as DIC, leave one out cross validation among others [51]. All the model selection criteria described in [51] are for non-spatial Bayesian models. These criteria are not suitable for validating a spatial model [50]. The reason of not considering any of these cross-validation techniques for model selection is that the spatial component of the model adds an additional objective that often competes with goodness-of-fit. That is, goodness-of-smoothing and goodness-of-fit criteria will often lead to different "best" models. Additionally, in a spatial setting for small area level data, leaving out one or more small areas from the model as in cross validation would completely change the neighbourhood patterns, and underlying spatial dependence. Thus the goodness of fit and model selection for spatial modelling needs different considerations [50]. So we followed the recommendations of Duncan and Mengersen [50] and chose WAIC as the model selection criteria, as it is showed valid in the context of spatial models as well. While this criteria is not perfect for spatial models, more research needs to be conducted to find out ways to apply cross validation or other selection criteria for Bayesian spatial models in small area level, which is beyond the scope of this current study.

On page 3, I can't see why the authors present the empirical likelihood ratio function R(F,w)=∏_(i=1)^n▒〖nw_i 〗 , where they mentioned w_i=1/n for all i

Response:

I agree there has been some confusion with multiple equations and multiple definitions on section 2, recap of the Bayesian Empirical Likelihood. In the current version, multiple formulations of EL and BEL is removed the section is reformatted including only the relevant formulations used in this article for better understanding to the readers.

Updated recap in section 2:

2 Background on BEL and SBEL Models

This section briefly discusses the background of Bayesian Empirical Likelihood (BEL) and SBEL models from the existing literature.

2.1 Recap of Bayesian Empirical Likelihood

Empirical likelihood (EL) combines the reliability of non-parametric methods with the flexibility and effectiveness of the likelihood approach. Owen, AB. [2] showed that the empirical likelihood ratio function can be used to construct confidence intervals for a sample mean, for a class of M-estimates and for other differentiable statistical functionals. EL can be constructed without any assumptions about the parametric family of distributions of the data and can be used for hypothesis testing as well as construction of confidence regions without an information or variance estimator. For data points $y_1, y_2, ..., y_n$ from some unknown distribution F, let some functional of F,

say $\boldsymbol{\theta}(F)$ be the parameter of interest for inference, which can be determined by the estimating equation $f(y_i, \boldsymbol{\theta})$, the EL function can be defined as [2]:

$$\hat{L}(F, w) = \prod_{i=1}^{n} w_i$$

where w_i satisfies the constraints $\sum_{i=1}^{n} w_i = 1, \sum_{i=1}^{n} w_i f_i(y_i, \boldsymbol{\theta}) = 0$.

Constrained optimization of the EL ratio function is carried out in order to obtain the EL weights w_i which are used as the data likelihood [24].

In a Bayesian framework, the likelihood is used to update a prior distribution and yield posterior inference. Lazar, N. [1] argued that EL can be used in place of a density and, when multiplied by the prior of the parameter of interest can yield the posterior distribution in such an analysis. The author explored the characteristics of Bayesian inference using EL instead of a parametric density. Starting from a well-known parametric case of a Bayesian posterior of a parameter vector $\boldsymbol{\theta}$, Scennach, S. M. [3] derived an EL posterior using the weights attributed to the sample points, which can be calculated by solving an entropy maximisation problem,

$$p(\boldsymbol{\theta}|Y) \propto p(\boldsymbol{\theta}) \prod_{i=1}^{n} w_i^*(\boldsymbol{\theta}) \tag{1}$$

where $p(\boldsymbol{\theta})$ is a given prior on $\boldsymbol{\theta}$ and $(w_1^*(\boldsymbol{\theta}), ..., w_n^*(\boldsymbol{\theta}))$ is the solution of:

$$argmax \sum_{i=1}^{n} -w_i \log(w_i), \text{subject to} \sum_{i=1}^{n} w_i = 1, \sum_{i=1}^{n} w_i f_i(y_i, \boldsymbol{\theta}) = 0 \tag{2}$$

where $f_i(y_i, \boldsymbol{\theta})$ represents the estimating equations of interest. The estimating equations can be formulated using the moment conditions [3,5].

On page 8, could authors give more explanation why SBEL approach is not able to provide enough information to sample the spatial dependence parameter ρ using MCMC since the inter-dependent nature of ψ and ρ does not seem convincing enough?

Response:

The parameters ψ (psi) and ρ (rho) are strongly interdependent, which results in a very difficult posterior space for the Random Walk Metropolis-Hastings to adequately explore and obtain independent Monte Carlo draws from. In our investigations of MCMC sampling with free psi and rho, we found that increases the MH kernel variance did not result in more independent

samples as even a small increase resulted in a very low acceptance rate for new parameter values. It is for this reason we decided to fix one parameter and sample the other. It is to be noted that in sampling spatial random effects, use of Gibbs sampling is predominant in the literature (Kang & Cressie,2011; Sengupta & Cressie, 2013). In SBEL approach, we cannot use Gibbs sampling due to lack of likelihood for the response variable. Hence, we decided to fix one parameter, rho and sample psi, the spatial random effects. This has now been added to section 3.2 of the paper:

The parameters ψ and ρ are strongly interdependent, which results in a very difficult posterior space for the Random Walk Metropolis-Hastings to adequately explore and obtain independent Monte Carlo draws from. In our investigations of MCMC sampling with free ψ and ρ , we found that increases the MH kernel variance did not result in more independent samples as even a small increase resulted in a very low acceptance rate for new parameter values. It is for this reason we decided to fix one parameter and sample the other. It is to be noted that in sampling spatial random effects, use of Gibbs sampling is predominant in the literature [41, 42]. In SBEL approach, we cannot use Gibbs sampling due to lack of likelihood for the response variable. Hence, we decided to fix one parameter, ρ and sample ψ, the spatial random effects.

A grid search approach is recommended to find an appropriate ρ using the SBEL-CAR model structure. The posterior samples under this model can be drawn for each of the proposed values of ρ and identify the most suitable ρ for a given dataset can be determined using a model performance criterion, such as WAIC [43, 44]. The fixed value of ρ is then used in the algorithm to sample the spatial random effects. Please see Appendix 2 for illustration.

In the section of "The Scottish Lip Cancer Data," author compared the results from parametric and semi-parametric models and found the semi-parametric model outperforms in terms of WAIC. In parametric model, they assumed that the response follows a normal distribution, but they also mentioned that alternative model, Poisson, might be possible choice for the analysis. I'm wondering if WAIC would suggest the parametric model in this analysis once Poisson is applied here.

Response:

For the section, "Scottish Lip Cancer Data", it is seen that the parametric models outperform the semiparametric model even when modelling the logarithm of SIR for each small area using each of the parametric models. This is because the Scottish Lip cancer data is a benchmark data where the log(SIR) follows a normal distribution (alternatively the count of deaths follow a Poisson distribution). So using this data, under normal or Poisson, parametric models are better fits to the data. Using WAIC, the Poisson models outperform the semiparametric models as well, similar to those we found in the analysis. This has been mentioned in the paper (section 4.1) as:

The comparative performance of the models is summarised in Table 1. It is observed that all the SBEL models provided similar estimates of posterior medians for fixed effects. However, in the SBEL models, the 95% credible intervals were wider than those of the parametric models. Hence the results obtained are encouraging as they show that the estimation for the parameters β made from SBEL-CAR models are similar to those obtained by using parametric models. It is noted that the Scottish lip cancer example is a benchmark data set for which parametric assumptions hold, so it is not surprising to see parametric models performing better according to WAIC and posterior distributions. In cases in which it is not straightforward to assume the parametric distribution, SBEL models can be useful to draw posterior inference and make predictions.

In the section of "Covid-19 data," this is a very interesting analysis. Is it possible that authors can provide the geographical distribution of some statistics from Covid-19 data like Figs A2 and A4; the discussion how to deal with the temporal correlation for the data; and the cluster (group, pattern) of observed data over time.

Response:

Spatial maps for observed deaths per million for the three periods are now added. The temporal correlation in the observed data and how to consider this in model is briefly mentioned with appropriate reference. The spatially smoothed deaths per million for each of the spatial models (parametric and SBEL) are added in the appendix as well.

Spatial map added to the section 4.4:

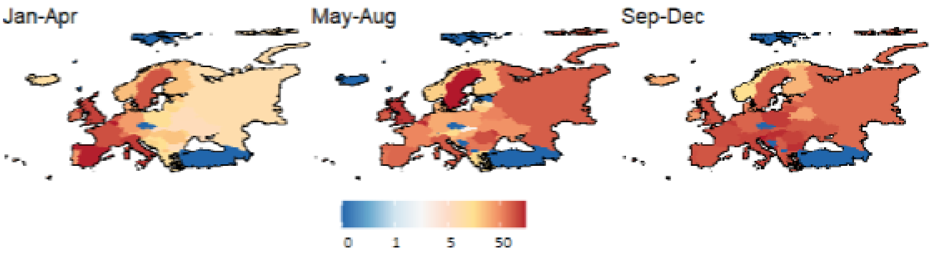

Fig 7. Observed new deaths per million due to COVID 19 in Europe, 2020

Brief discussion on temporal correlation and pattern added to 4.4

The variation in observed deaths per million in Europe is visible in the three time periods of 2020. To account for the temporal correlation, one option is to add a temporal component using a linear time trend, along with a spatial-temporal interaction term [58]. In the present article, we are concentrating on spatial models only and ignoring the temporal component. Adding temporal component and spatial temporal interaction might improve the model performance, which can be checked as a future extension of this study. The Fig. 7 also exhibits some groups of countries having consistently lower numbers of deaths over the 3 periods, some of the countries show increase or decrease over time. More investigation is possible using the dataset in order to determine clusters and patterns of observed deaths over time, which is beyond the scope and focus of this current study.

Spatial map added to the appendix:

The numerical values of the posterior summaries using each model for periods 1, 2 and 3 are shown in Table A1 in Appendix 1. The spatially smoothed values for new deaths per million in Europe for the period 3(Sep-Dec) are shown in Fig. A5 (Appendix 1).

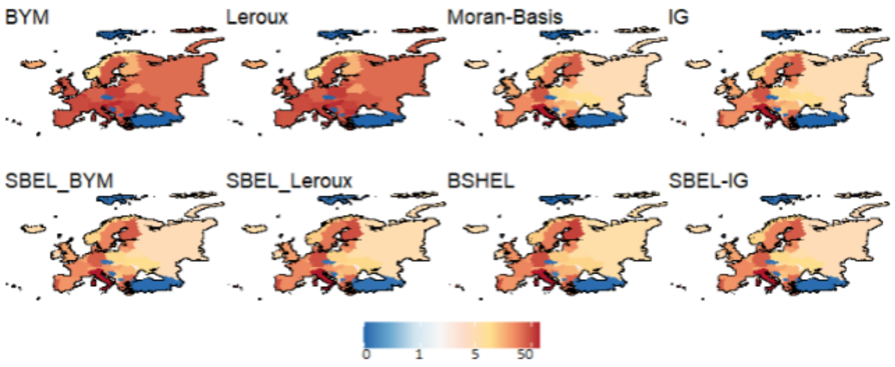

Fig.A5: Smoothed new deaths per million for Europe in Sep-Dec, 2020.

Typo: (I list some)

On page 3, line 71: Constrained optimization of the EL ratio function “is” done in order …

On page 8, line 242: a model to with …

On page 8, line 260: “Porter” et al.

On page 8, Equation (16) should be added “,” before “where g represents…”

On page 10, line 310, \phi^*{(t-1)} is the value of \phi^* in the previous …

Response:

Thanks for pointing out the typos. The mentioned ones and some others are corrected now in the revised version.

Response to Reviewer 2:

Comments

This article is poorly written. It is unable to deliver the main idea of the proposed model that employs the empirical likelihood (Section 2.1), so it seems that the proposed model is a regular Bayesian model with implementation with Gibbs sampler (Section 3.2).

Response:

The main idea of the paper was to extend the use of Bayesian Empirical Likelihood framework in modelling small area level data. In spite of usefulness of such semiparametric approaches of spatial modelling, there has not been much literature in this field. So the present article contributes to the literature of spatial BEL models using detailed algorithm and real life case studies.

1. The recaps (Section 2) are poorly written, this section is not able to point out the main idea of the empirical likelihood. I think the authors might want to skip the whole section. Here for example in page 3 line 69, it mentions that w_i = 1nfor all i, not sure whether it is the same as in [24], but in line 83, it suggests that this w_i could be found in [24] and not sure how we could derive from the equations in line 80. Furthermore, in equation 1, w_i turns out to be $w_i(q)$, and not sure how this connects to the previous equations in line 80. And in equation 2, the w_is now are not related to q. So, I am not sure how the readers could go further in next section.

Response:

The whole point of section 2 was to give an idea of EL and BEL as it is used in spatial modelling in the data stage of Bayesian hierarchical framework. The purpose of the recap is not to discuss on importance and main idea of Empirical likelihood, rather it is introducing the Empirical likelihood to use the BEL in spatial modelling. There have been heaps of literature discussing the main idea and significance of Empirical Likelihood, that was not the intention of this section.

However, as commented, there has been some issues and confusion with many equations and notations. In the current version, multiple formulations of EL function and BEL is given which might be confusing to the readers. So, the equations, notations are updated and the section is reformatted including only the relevant formulations used in this article for clarity.

2 Background on BEL and SBEL Models

This section briefly discusses the background of Bayesian Empirical Likelihood (BEL) and SBEL models from the existing literature.

2.1 Recap of Bayesian Empirical Likelihood

Empirical likelihood (EL) combines the reliability of non-parametric methods with the flexibility and effectiveness of the likelihood approach. Owen, AB. [2] showed that the empirical likelihood ratio function can be used to construct confidence intervals for a sample mean, for a class of M-estimates and for other differentiable statistical functionals. EL can be constructed without any assumptions about the parametric family of distributions of the data and can be used for hypothesis testing as well as construction of confidence regions without an information or variance estimator. For data points $y_1, y_2, ..., y_n$ from some unknown distribution F, let some functional of F,

say $\boldsymbol{\theta}(F)$ be the parameter of interest for inference, which can be determined by the estimating equation $f(y_i, \boldsymbol{\theta})$, the EL function can be defined as [2]:

$$\hat{L}(F, w) = \prod_{i=1}^{n} w_i$$

where w_i satisfies the constraints $\sum_{i=1}^{n} w_i = 1, \sum_{i=1}^{n} w_i f_i(y_i, \boldsymbol{\theta}) = 0$.

Constrained optimization of the EL ratio function is carried out in order to obtain the EL weights w_i which are used as the data likelihood [24].

In a Bayesian framework, the likelihood is used to update a prior distribution and yield posterior inference. Lazar, N. [1] argued that EL can be used in place of a density and, when multiplied by the prior of the parameter of interest can yield the posterior distribution in such an analysis. The author explored the characteristics of Bayesian inference using EL instead of a parametric density. Starting from a well-known parametric case of a Bayesian posterior of a parameter vector $\boldsymbol{\theta}$, Scennach, S. M. [3] derived an EL posterior using the weights attributed to the sample points, which can be calculated by solving an entropy maximisation problem,

$$p(\boldsymbol{\theta}|Y) \propto p(\boldsymbol{\theta}) \prod_{i=1}^{n} w_i^*(\boldsymbol{\theta}) \quad (1)$$

where $p(\boldsymbol{\theta})$ is a given prior on $\boldsymbol{\theta}$ and $(w_1^*(\boldsymbol{\theta}), ..., w_n^*(\boldsymbol{\theta}))$ is the solution of:

$$argmax \sum_{i=1}^{n} -w_i \log(w_i), \text{subject to} \sum_{i=1}^{n} w_i = 1, \sum_{i=1}^{n} w_i f_i(y_i, \boldsymbol{\theta}) = 0 \quad (2)$$

where $f_i(y_i, \boldsymbol{\theta})$ represents the estimating equations of interest. The estimating equations can be formulated using the moment conditions [3,5].

2. This article focus on the spatial model and here I could only see the priors available in section 3. Where I could see the spatial effect contributed by the empirical or it is just a simple extension?

Response:

The focus of this paper is to use Empirical likelihood and spatial priors to create a semiparametric approach of modelling small area level data. The paper is outlined to give the readers a background of both empirical likelihood and spatial priors before the model formulated in section 3. The recap on priors are also located in section 2 along with the review of BEL models.

The spatial component is not contributed by the empirical likelihood, as it has been clearly mentioned in the paper, rather this is extension of Bayesian hierarchical modelling approach using Empirical Likelihood at data stage and spatial priors in parameter stage of the model. So yes, this is an extension of the work Porter et al. (2015) but not a simple extension. It was challenging to extend the BEL models to employ the CAR spatial priors, which has not been done before in any literature, to the best of our knowledge. There were computational challenges as well. Also this paper investigates in detail the usefulness of the spatial BEL models in benchmark datasets, simulated datasets and recent COVID19 dataset. So in our

opinion, this paper can contribute to the spatial modelling literature avoiding the data distribution by replacing data likelihood by Empirical Likelihood.

3. Here I could see the likelihood in equations 23, 24 I guess, that is p(Yjy;b) and is it a component in the empirical likelihood?

Response:
Yes, in equation 23, 24, to define the posterior density ratio, $p(Y_i|y;b)$ is used which is replaced by the empirical likelihood weights in next sentence. The usual definition of posterior density ratio includes likelihood multiplied by the prior, but in this stage data likelihood is replaced by the Empirical likelihood. We have followed the standard notations used in Porter et al. (2015).

4. In the simulation study, I am not sure why the posterior median are given, not posterior mean (commonly used) in Tables 1, 2.

Response:
Posterior mean and median both are calculated, the posterior medians were chosen in order to provide more robust measure. But according to the reviewer's comment, we have replaced it with Posterior means. It does not change the results and interpretation.

5. I think the contribution of this article is quite limited.

Response:

The purpose of the paper is to show in detail when and where spatial BEL models can be effective in modelling small area level data using simulation and real-life data, that was not present in any of the previous literature before. The article creates scope for further research in spatial modelling using Bayesian Empirical Likelihood in modelling irregular data in small area level. It also gives opportunities to extend the Bayesian hierarchical framework using EL and different classes of spatial priors to get detailed knowledge of impact on model performance for different priors under BEL framework.
