# Supplementary material for: Evaluation of spatial Bayesian Empirical Likelihood models in analysis of small area data
Source: PLoS One. 2022 May 27;17(5):e0268130. doi: 10.1371/journal.pone.0268130 (PMC9140259; doi:10.1371/journal.pone.0268130)
Supplement: Attachment — Submitted filename: Response to reviewers comment.pdf [file pone.0268130.s004.pdf]

Reviewer’s comment:

The authors have addressed the issues I raised. But the recaps are not well written so it is somewhat hard for us to capture the main idea of the empirical likelihood.

Response:

Thanks for reviewing the manuscript. In order to improve the recap of empirical likelihood, revision is made by creating two subsections on Empirical likelihood (2.1.1) and Bayesian Empirical Likelihood (2.1.2). Both these subsections now contain more information with additional references for better understanding of EL and BEL. Since there is sufficient existing literature already to explain the main idea, scope and benefits of EL, we did not elaborate this on the paper, rather we focused on using the BEL for modelling the small area level data.

The revised section in the paper is as follows:

2.1 Recap of Bayesian Empirical Likelihood

2.1.1 Empirical Likelihood

Empirical likelihood (EL) combines the reliability of non-parametric methods with the flexibility and effectiveness of the likelihood approach. To overcome the model misspecification and lack of robustness of parametric likelihood, a non parametric analogue of parametric likelihood, empirical likelihood (EL) was introduced by Owen [2]. Owen [2] initially showed that the empirical likelihood ratio function can be used to construct confidence intervals for a sample mean, for a class of M-estimates and for other

differentiable statistical functional. These methods were framed as a non-parametric extension of Wilk’s [24] theorem for parametric likelihood ratio tests. Later EL was expanded to all types of estimating equations by Qin and Lawless [25].

For data points $y_1, y_2, ..., y_n$ from some unknown distribution F, let some functional of F, say $\boldsymbol{\theta}(F)$ be the parameter of interest for inference, which can be determined by the estimating equation $f(y_i, \boldsymbol{\theta})$, the EL function can be defined as [26]:

$$\hat{L}(F, w) = \prod_{i=1}^{n} w_i$$

where w_i satisfies the constraints $\sum_{i=1}^{n} w_i = 1, \sum_{i=1}^{n} w_i f_i(y_i, \boldsymbol{\theta}) = 0$.

Constrained optimisation of the EL ratio function is carried out in order to obtain the EL weights w_i which are used as the data likelihood [26].

The introduction to estimating equations to EL enhanced the scope of EL to so many different applications including generalised linear models [27], time series [7, 28], econometrics [29], regression analysis [30, 31], survival analysis [32] and many more. For more details on scope and benefits of EL, we refer the reader to Lazar [33], which reviewed EL from its initiation to current developments in theory and applications along with the potential for future development.

2.1.2 Bayesian Empirical Likelihood

In a Bayesian framework, the likelihood is used to update a prior distribution and yield posterior inference. Lazar [1] argued that EL can be used in place of a density and, when multiplied by the prior of the parameter of interest can yield the posterior distribution in such an analysis. The author explored the characteristics of Bayesian inference using EL instead of a parametric density. Starting from a well-known parametric case of a Bayesian posterior of a parameter vector $\boldsymbol{\theta}$, Schennach [3] derived an EL posterior using the weights attributed to the sample points, which can be calculated by solving an entropy maximisation problem,

$$p(\boldsymbol{\theta}|Y) \propto p(\boldsymbol{\theta}) \prod_{i=1}^{n} w_i^*(\boldsymbol{\theta}) \tag{1}$$

where $p(\boldsymbol{\theta})$ is a given prior on $\boldsymbol{\theta}$ and $(w_1^*(\boldsymbol{\theta}), ..., w_n^*(\boldsymbol{\theta}))$ is the solution of:

$$argmax \sum_{i=1}^{n} -w_i \log(w_i), \text{subject to} \sum_{i=1}^{n} w_i = 1, \sum_{i=1}^{n} w_i f_i(y_i, \boldsymbol{\theta}) = 0 \tag{2}$$

where $f_i(y_i, \boldsymbol{\theta})$ represents the estimating equations of interest. The estimating equations can be formulated using the moment conditions [3,5].

Schennach [3] showed that for large enough samples, BEL offered similar results to Bayesian bootstrap. Grendar and Judge [34] demonstrated that the BEL is an asymptotic approximation of the Bayesian maximum a posteriori probability estimators, which provided additional justification of using EL in a Bayesian setting [33]. As a result, BEL has been applied in quantile regression [35], ridge and lasso regression [36], inference with complex survey data [37], spatial data analysis [10–12,38] and so on. The spatial analysis using BEL is the focus of this study.
